# Diagnostic Accuracy of International Classification of Diseases, Tenth Revision (ICD-10) Codes for Vertebral Artery Dissection and Its Complications in Japan: A Single-Center Validation Study

**DOI:** 10.7759/cureus.108794

**Published:** 2026-05-13

**Authors:** Yuji Okazaki, Yuki Kataoka, Toshihisa Ichiba

**Affiliations:** 1 Systematic Reviews, Scientific Research Works Peer Support Group (SRWS-PSG), Osaka, JPN; 2 Emergency Medicine, Hiroshima City Hiroshima Citizens Hospital, Hiroshima, JPN; 3 Healthcare Epidemiology, School of Public Health, Kyoto University Graduate School of Medicine, Kyoto, JPN; 4 Center for Postgraduate Clinical Training and Career Development, Nagoya University Hospital, Nagoya, JPN; 5 Center for Medical Education, Graduate School of Medicine, Nagoya University, Nagoya, JPN; 6 International and Community Oral Health, Tohoku University Graduate School of Dentistry, Sendai, JPN; 7 Internal Medicine, Kyoto Min-iren Asukai Hospital, Kyoto, JPN

**Keywords:** acute cerebral infarction, japanese medical claims data, spontaneous vertebral artery dissection, subarachnoid hemorrhage, validation study

## Abstract

Introduction: Vertebral artery dissection (VAD) is a leading cause of cerebrovascular disease in young and middle-aged adults. Although medical claims databases facilitate large-scale observational studies, validated case-identification algorithms for unruptured VAD and its complications remain limited. We evaluated the diagnostic accuracy of International Classification of Diseases, Tenth Revision (ICD-10) codes for identifying unruptured VAD and its complications in Japanese medical claims data.

Methods: We conducted a single-center cross-sectional validation study using electronic medical records from April 2018 to December 2025. Three ICD-10-based case definitions were evaluated: I72.6 for unruptured VAD, I60.5 for VAD-related subarachnoid hemorrhage (SAH), and the combination of I72.6 with I63.0-I63.9 for unruptured VAD with cerebral infarction. Two independent clinicians determined reference standards based on imaging findings and documented clinical diagnoses. The primary outcome was a positive predictive value (PPV), with 95% confidence intervals (CI) calculated using the Wilson method.

Results: A total of 104 patients met the ICD-10-based case definitions (64 with I72.6, 12 with I60.5, and 28 with combined I72.6 and I63.0-I63.9). The PPV was 0.81 (95% CI, 0.70-0.89) for I72.6 alone, 0.82 (95% CI, 0.55-0.95) for I60.5, and 1.00 (95% CI, 0.88-1.00) for the combined I72.6 and I63.0-I63.9. When I72.6 was restricted to codes assigned on the hospital admission date, the PPV increased to 0.93 (95% CI, 0.81-0.98).

Conclusion: The combination of ICD-10 codes for unruptured VAD and cerebral infarction yielded a high PPV and may be suitable as a pragmatic initial case identification algorithm for pharmacoepidemiologic studies using Japanese medical claims databases.

## Introduction

Vertebral artery dissection (VAD) is a leading cause of cerebrovascular diseases among young and middle-aged adults, particularly in Asian populations, including Japan [1,2]. Despite advances in imaging modalities, early diagnosis remains challenging because clinical presentations of VAD are often non-specific [3]. In addition, VAD may lead to life-threatening complications, including subarachnoid hemorrhage (SAH) or cerebral infarction, often within days of symptom onset, and both are associated with a poor prognosis [3,4].

Although substantial research has investigated the diagnosis and management of VAD, evidence remains conflicting [5], resulting in variation in clinical practice among clinicians [6]. Because randomized trials may not reflect the clinical heterogeneity and varied practice patterns in real-world settings, large-scale observational studies may help address these unresolved issues regarding VAD and its complications. Medical claims databases provide opportunities to conduct such studies [7]. However, the validity of such observational studies critically depends on the accurate identification of target conditions. Researchers have not yet established validated International Classification of Diseases, Tenth Revision (ICD-10) codes or diagnostic algorithms for identifying unruptured VAD and its complications [8].

To address this research gap, we aimed to investigate the diagnostic accuracy of ICD-10 codes for unruptured VAD, VAD-related SAH, and unruptured VAD with cerebral infarction in Japanese medical claims data.

## Materials and methods

Study design and settings

We conducted this cross-sectional study using electronic medical record data from Hiroshima City Hiroshima Citizens Hospital between April 1, 2018, and December 31, 2025. This study was approved by the Institutional Review Board of Hiroshima City Hiroshima Citizens Hospital (approval no. 2025-150). The necessity for obtaining informed consent was omitted due to the anonymized nature of the data, and an opt-out strategy was implemented for participant recruitment. We adhered to reporting guidelines for assessing the quality of validation studies of health administrative data (Appendix 1) [9].

Study participants

We included all patients aged 18 years or older who visited our hospital between April 1, 2018, and December 31, 2025, regardless of outpatient or inpatient status. Patients were considered potentially eligible if they had ICD-10 codes, as defined by the World Health Organization, indicative of unruptured VAD, VAD-related SAH, or unruptured VAD with cerebral infarction. Eligible cases were identified using ICD-10 codes (I72.6, I60.5, or I63.0-I63.9) recorded in either outpatient claims data or inpatient medical claim Form 1 (Table 1). We did not exclude patients with out-of-hospital or in-hospital cardiac arrest. Patients were excluded if they had traumatic or iatrogenic vertebral artery injuries identified by the following ICD-10 codes: S15.1, T81.2, and T81.7 (Appendix 2). For patients with recurrent VAD who were assigned ICD-10 codes at different time points, only the first episode was included in the analysis. The unit of analysis was the individual patient.

**Table 1:** ICD-10 codes for vertebral artery dissection and its complications in Japanese claim data ICD-10: International Classification of Diseases, Tenth Revision

Complications	ICD-10 codes
(1) Unruptured vertebral artery dissection or aneurysm	I72.6
(2) Vertebral artery dissection-related subarachnoid hemorrhage (SAH) or vertebral artery aneurysm-related SAH	I60.5
(3) Unruptured vertebral artery dissection with cerebral infarction	I72.6 + I63.0, I63.1, I63.2, I63.3, I63.4, I63.5, I63.6, I63.7, I63.8, I63.9

Target conditions

The target conditions in this study were: unruptured intracranial or extracranial VAD, VAD-related SAH, and unruptured VAD with cerebral infarction [5].

Index test

We defined the index test as positive when the following ICD-10 codes were recorded as outpatient claims data or either primary or secondary diagnoses on inpatient medical claim Form 1: I72.6 for unruptured VAD, I60.5 for VAD-related SAH, and I72.6 combined with codes I63.0 through I63.9 for unruptured VAD with cerebral infarction (Algorithm 1). For unruptured VAD only, we additionally defined Algorithm 2, which used the same definition as Algorithm 1 with an additional restriction: code I72.6 was considered positive only when the code assignment date coincided with the hospital admission date.

Reference standard

We defined the reference standard for each of the three target conditions as follows. For unruptured VAD, the reference standard was considered positive when both of the following were met: radiologists, neurologists, or neurosurgeons diagnosed VAD based on imaging findings, including intramural hematoma on T1-weighted head magnetic resonance imaging (MRI); pearl-and-string sign on head magnetic resonance angiography (MRA); eccentric or crescent-shaped vessel-wall thickening on head computed tomography angiography (CTA); or intimal flap, double lumen, or dissecting aneurysm on cerebral angiography [5,10]; and a neurologist or neurosurgeon explicitly documented the diagnosis of VAD in the medical records. For VAD-related SAH, the reference standard was considered positive when all of the following were met: VAD was diagnosed on imaging as described above; a neurologist or neurosurgeon explicitly documented the diagnosis in the medical records; and imaging findings consistent with SAH were present on head CT. For unruptured VAD with a cerebral infarction, the reference standard was considered positive when all of the following were met: VAD was diagnosed on imaging as described above; a neurologist or neurosurgeon explicitly documented the diagnosis in the medical records; and imaging findings consistent with cerebral infarction were present on brain MRI. Regarding the time lag between ICD-10 code assignment (index test) and imaging examination date (reference standard), the following periods were established for each of the three target conditions, regardless of the sequence of index test and reference standard: unruptured VAD: one day, ruptured VAD-related SAH: one day, and unruptured VAD with cerebral infarction: 14 days [11,12]. This reference was determined standard through electronic medical record review by two independent clinicians (YO and TI). Discrepancies between the two reviewers regarding positive reference standards were resolved through discussion to reach a final determination.

Outcomes

The primary outcome was the positive predictive value (PPV) of ICD-10 codes for diagnosing the target conditions related to VAD. We calculated PPV for each of the three target conditions.

Statistical analysis

We summarized descriptive statistics as frequencies (%) for binary and categorical variables and as medians and interquartile ranges (IQR) for continuous variables. We calculated PPV as (number of cases confirmed as true positive by reference standard [true positive]) / (number of cases positive by index test [true positive + false positive]). The 95% confidence intervals (CIs) for PPV were calculated using the Wilson score method. We performed statistical analyses using R software (version 4.4.3; R Foundation for Statistical Computing, Vienna, Austria).

## Results

Study participants characteristics

**Figure 1:**
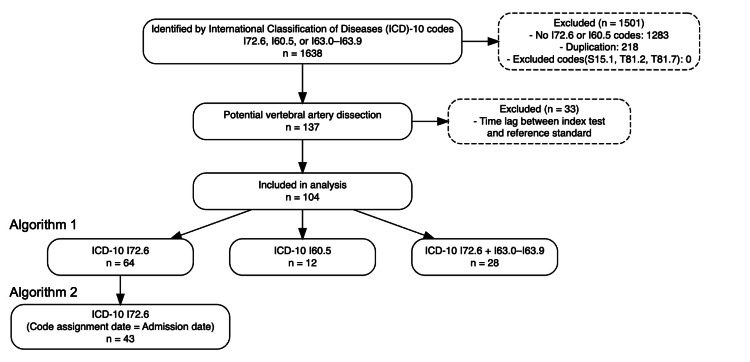
Flow diagram of ICD-10-based case identification between April 1, 2018, and December 31, 2025 ICD-10: International Classification of Diseases, Tenth Revision ICD-10 code I72.6 was used to identify patients with unruptured vertebral artery dissection (VAD) or aneurysm. Code I60.5 identified patients with VAD-related subarachnoid hemorrhage (SAH) or vertebral artery aneurysm-related SAH. Code I72.6, in combination with codes I63.0-I63.9, identified patients with unruptured VAD with cerebral infarction. Code S15.1 identified patients with traumatic vertebral artery injury. Codes T81.2 and T81.7 were used to identify iatrogenic vascular injury.

Using Algorithm 1, we included 104 patients in our analysis: 64 (62%) with code I72.6, 12 (12%) with code I60.5, and 28 (27%) with codes I72.6 combined with I63.0-I63.9 (Figure 1).

Algorithm 2 included 43 (67%) patients with code I72.6. Thirty-three patients (24%) were excluded due to an excessive time lag between the index test and reference standard (Table 2).

**Table 2:** Distribution of time lag between index test and reference standard in excluded patients Data is presented as n (%).

Time lag (days)	I72.6 (n=31)	I60.5 (n=1)	I72.6 + I63.0-I63.9 (n=1)
2	1 (3.2)	-	-
3	2 (6.5)	-	-
4	1 (3.2)	-	-
5	1 (3.2)	-	-
6	1 (3.2)	-	-
7	3 (9.7)	-	-
8	1 (3.2)	-	-
9	4 (12)	-	-
10	2 (6.5)	-	-
12	2 (6.5)	-	-
14	3 (9.7)	-	-
15	-	-	1 (100)
20	1 (3.2)	1 (100)	-
21	1 (3.2)	-	-
>30	7 (23)	-	-
Not applicable	1 (3.2)	-	-

The majority of included patients were middle-aged males (Table 3).

**Table 3:** Characteristics of ICD-10 codes-identified patients ICD-10: International Classification of Diseases, Tenth Revision; IQR: interquartile range * When patients underwent multiple diagnostic tests, we selected all applicable tests. † Magnetic resonance angiography was included.

Characteristics	I72.6 (n=64)	I60.5 (n=12)	I72.6 + I63.0-I63.9 (n=28)
ICD-10 codes, n (%)			
I72.6 + I63.3	-	-	7 (25)
I72.6 + I63.5	-	-	14 (50)
I72.6 + I63.8	-	-	2 (7.1)
I72.6 + I63.9	-	-	5 (18)
Age (years), median (IQR)	52 (42, 56)	55 (52, 65)	48 (44, 56)
Male, n (%)	35 (55)	7 (58)	20 (71)
Diagnostic test^*^, n (%)			
Magnetic resonance imaging^†^	62 (97)	0	28 (100)
Computed tomography angiography	2 (3.1)	12 (100)	0
Cerebral angiography	4 (6.3)	10 (83)	4 (14)

Positive predictive values of ICD-10 codes for VAD and its complications

Table 4 presents the PPVs of ICD-10 codes for VAD and its complications. Using Algorithm 1, the PPV was 0.81 (95% CI: 0.70-0.89) for code I72.6, 0.82 (95% CI: 0.55-0.95) for code I60.5, and 1.0 (95% CI: 0.88-1.0) for codes I72.6 combined with I63.0-I63.9. Using Algorithm 2, the PPV improved to 0.93 (95% CI: 0.81-0.98).

**Table 4:** Positive predictive value of ICD-10 codes for vertebral artery dissection and its complications PPV: positive predictive value, CI: confidence interval; VAD: vertebral artery dissection; SAH: subarachnoid hemorrhage; ICD-10: International Classification of Diseases, Tenth Revision * Algorithm 1 included patients with ICD-10 codes I72.6 (unruptured VAD), I60.5 (VAD-related SAH), or I72.6 combined with I63.0-I63.9 (unruptured VAD with cerebral infarction) recorded as primary or secondary diagnoses on medical claim Form 1. † Algorithm 2 applied the same definition as Algorithm 1 for code I72.6, with an additional restriction requiring the code assignment date to coincide with the hospital admission date.

	Algorithm 1^*^			Algorithm 2^†^		
Index test	Total number	True positive	PPV (95% CI)	Total number	True positive	PPV (95% CI)
I72.6	64	52	0.81 (0.70 - 0.89)	43	40	0.93 (0.81 - 0.98)
I60.5	12	10	0.82 (0.55 - 0.95)			
I72.6 + I63.0-I63.9	28	28	1.0 (0.88 - 1.0)			

## Discussion

This Japanese ICD-10-based validation study found that the combination of code I72.6 with cerebral infarction codes (I63.0-I63.9) yielded a high PPV for identifying unruptured VAD with cerebral infarction. In contrast, code I72.6 alone for unruptured VAD and code I60.5 for VAD-related SAH showed only moderate PPVs. However, the PPV for unruptured VAD improved when the assignment date of code I72.6 coincided with the hospital admission date.

The high PPV for unruptured VAD with cerebral infarction likely reflects the increased specificity achieved by requiring co-occurrence of an unruptured VAD code and a cerebral infarction code, which narrows the case definition and reduces false-positive identification. In contrast, the moderate PPVs for I72.6 alone and I60.5 would result from the limited specificity of these codes, which can capture vertebral artery aneurysms in addition to dissection. Restricting I72.6 to cases assigned on the hospital admission date improved the PPV, and further refinement incorporating repeated diagnostic codes, procedure codes, or imaging-related records might enhance identification accuracy for these conditions.

The present study addresses an important gap in Japanese claims-based research on VAD. Prior validation studies in Japan have established ICD-10-based diagnostic algorithms for other cardiovascular and cerebrovascular conditions, including acute coronary syndrome, heart failure, aortic disease [13], and acute ischemic stroke [14]. However, to our knowledge, no study has validated ICD-10 codes for unruptured VAD or its complications in Japanese claims data. Without such validation, the reliability of claims-based epidemiological studies on VAD remains uncertain. Our findings provide an empirical foundation for selecting ICD-10 codes to identify VAD and its complications in Japanese claims databases.

This study has several limitations, and our findings should be carefully interpreted. First, this study was conducted at a single center with a small sample size. The generalizability of our findings may be limited. To overcome this limitation, multi-center large-scale studies are needed to obtain more precise estimates and to externally validate our findings. Second, we did not evaluate the sensitivity of ICD-10 codes for target conditions. As a result, the number of true patients with target conditions who were not captured at our institution remains unknown. To estimate sensitivity, future studies should identify true cases independently of ICD-10 codes, for example, by systematically reviewing discharge summaries, emergency department records, or radiology reports for imaging-confirmed VAD and then determining how many of these cases were assigned the corresponding ICD-10 codes. Third, inter-rater reliability for the reference standard diagnoses was not formally assessed, and the number of discrepancies between the two reviewers was not recorded. Therefore, the reliability of the reference standard could not be established. Furthermore, the reference standard used in this study may have limited applicability to future research, which should be considered when interpreting the PPV calculations.

## Conclusions

Our findings suggest that the combination of ICD-10 codes for unruptured VAD and cerebral infarction shows high precision in identifying this condition in Japanese medical claims databases and may serve as a reasonable starting point for pharmacoepidemiologic studies on VAD. However, as sensitivity was not assessed in this study, the completeness of case identification remains uncertain. In contrast, unruptured VAD and VAD-related SAH identified by single ICD-10 codes may not be suitable for such studies. Further large-scale studies are needed to confirm our findings.

## Appendices

Appendix 1

**Table 5:** The checklist of reporting guidelines for assessing the quality of validation studies of health administrative data

	YES	NO	UNCERTAIN	NOT APPLICABLE
TITLE, KEYWORDS, ABSTRACT				
Identify article as study of assessing diagnostic accuracy	○			
Identify article as study of administrative data	○			
INTRODUCTION:				
State disease identification & validation one of goals of study	○			
METHODS:				
Participants in validation cohort:				
Describe validation cohort (Cohort of patients to which reference standard was applied)	○			
∙ Age ∙ Disease ∙ Severity ∙ Location/Jurisdiction				
Describe recruitment procedure of validation cohort	○			
∙ Inclusion criteria ∙ Exclusion criteria				
Describe patient sampling (random, consecutive, all, etc.)	○			
Describe data collection	○			
∙ Who identified patients and did selection adhere to patient recruitment criteria ∙ Who collected data ∙ A priori data collection form ∙ Disease classification ∙ Split sample (i.e. re-validation using a separate cohort)				
Test Methods:				
Describe number, training and expertise of persons reading reference standard	○			
If >1 person reading reference standard, quote measure of consistency (e.g. kappa)				
Blinding of interpreters of reference standard to results of classification by administrative data e.g. Chart abstractor blinded to how that chart was coded	○			
Statistical Methods:				
Describe methods of calculating/comparing diagnostic accuracy	○			
RESULTS:				
Participants:				
Report when study done, start/end dates of enrollment	○			
Describe number of people who satisfied inclusion/exclusion criteria	○			
Study flow diagram	○			
Test results:				
Report distribution of disease severity	○			
Report cross-tabulation of index tests by results of reference standard	○			
Estimates:				
Report at least 4 estimates of diagnostic accuracy	○			
Diagnostic Accuracy Measures Reported: ∙ Sensitivity ∙ Specificity ∙ PPV ∙ NPV ∙ Likelihood ratios ∙ Kappa ∙ Area under the ROC curve / c-statistic ∙ Accuracy/agreement ∙ Other (specify)	○			
Report accuracy for subgroups (e.g. age, geography, different sex, etc.)		○		
If PPV/NPV reported, ratio of cases/controls of validation cohort approximate prevalence of condition in the population		○		
Report 95% confidence intervals for each diagnostic measure	○			
DISCUSSION:				
Discuss the applicability of the validation findings	○			

Appendix 2

**Table 6:** Excluded ICD-10 codes for identifying the target conditions ICD: International Classification of Diseases

	ICD-10 codes
Traumatic vertebral artery injury	S15.1
Iatrogenic vascular injury	T81.2 (Catheter-related vascular injury) T81.7 (Post-procedure vascular injury)
